# COPING VARIABILITY AND ITS ASSOCIATION WITH ALL-CAUSE MORTALITY

**DOI:** 10.1093/geroni/igac059.583

**Published:** 2022-12-20

**Authors:** Victoria Marino, Avron Spiro, Lewina Lee

**Affiliations:** Boston University School of Medicine, Boston, Massachusetts, United States; Boston University, Jamaica Plain, Massachusetts, United States; Boston University School of Medicine, Boston, Massachusetts, United States

## Abstract

Coping strategies – cognitive, behavioral, and emotional tactics used to manage stressors – are associated with morbidity and mortality. Between-strategy coping variability, defined as (un)evenness in usage across coping strategies, may reflect context-specific coping and account for additional variance in health outcomes beyond mean strategy use. This study examined prospective associations of mean coping strategy use and between-strategy coping variability with time to death in 823 men from the Normative Aging Study. In Cox proportional hazard models, 1-SD higher in mean usage of positive action, negative action, prayer, withdrawal, and substance use strategies was associated with 17-32% greater risk of all-cause mortality over 27 years, after adjusting for baseline demographics, health status, and depression. Contrary to prior research, mortality risk did not differ by coping variability. We will consider findings within the stress and coping framework and discuss implications for biobehavioral pathways linking coping to all-cause mortality.

